# Data supporting Arf6 regulation of Schwann cell differentiation and myelination

**DOI:** 10.1016/j.dib.2015.09.025

**Published:** 2015-10-03

**Authors:** Tomohiro Torii, Yuki Miyamoto, Masahiro Yamamoto, Katsuya Ohbuchi, Hideki Tsumura, Kazuko Kawahara, Akito Tanoue, Hiroyuki Sakagami, Junji Yamauchi

**Affiliations:** a Department of Pharmacology, National Research Institute for Child Health and Development, Setagaya, Tokyo 157-8535, Japan; b Department of Animal Facility, National Research Institute for Child Health and Development, Setagaya, Tokyo 157-8535, Japan; c Tsumura Research Laboratories, Tsumura & Co., Inashiki, Ibaraki 200-1192, Japan; d Department of Anatomy, Kitasato University School of Medicine, Sagamihara, Kanagawa 252-0374, Japan; e Graduate School of Medical and Dental Sciences, Tokyo Medical and Dental University, Bunkyo, Tokyo 113-8510, Japan

**Keywords:** Arf6, Schwann cell, Nerve, Myelination, Signaling

## Abstract

The data is related to the research article entitled “Arf6 mediates Schwann cell differentiation and myelination” [1]. To further investigate the role of Arf6 in promoting myelination by Schwann cells *in vivo*, we have characterized an another line (#2) of small-hairpin (sh)RNA transgenic mice targeting Arf6. The number of transgenes per one allele in this line was very low (2 transgenes), comparing with high copies in the previous line (#1, 20 transgenes) [1]. In 4 days of neonatal age, transgenic mice exhibited decreased myelin thickness; however, decreased levels were not as much as those in the line #1, likely depending on transgene copy number. In 60-day-old mice, the difference became smaller. On the other hand, transgene׳s effect was not related to cell proliferation and apoptosis. These data support the key role of Arf6 in Schwann cell myelination, especially in the initiation.

**Specifications table**

**Table:** 

Subject area	Biology
More specific subject area	Neurobiology, Molecular and cellular neuroscience, Cell and developmental biology
Type of data	Figure
How data was acquired	Immunoblotting, electron microscopy, and immunohistochemistry
Data format	Raw and analyzed data
Experimental factors	The *g*-ratios (the numerical ratios of the axon diameter to the diameter of the outermost myelinated fibers) for analyzing myelin thickness
Experimental features	Immunoblot and electron or fluorescent microscopic analysis
Data source location	National Research Institute for Child Health and Development, Tokyo, Japan
Data accessibility	Data is available with this study

**Value of the data**

• The data from *g*-ratios, the numerical ratios of the axon diameter to the diameter of the outermost myelinated fibers, provide the difference between transgenic mice and the littermate controls.
• The average *g*-ratios from neonatal mice support that Arf6 preferentially regulates the initiation of myelination.
• The effect is not responsible for cell growth and apoptosis.

## 1 Data, experimental design, materials and methods

### 1.1 Generation of Arf6 shRNA transgenic mouse

Mouse Arf6 shRNA oligonucleotides, corresponding to the siRNA target sequences (5′-AAGAATATCAGCTTCACCGTG-3′ and 5′-AAGATCCGGCCGCTCTGGCGG-3′), were inserted into the BLOCK-iT PolII-miR-RNAi expression vector (Life Technologies), followed by amplification with the 704-2010 bases. The MPZ promoter (GenBank Acc. No. M62857) for Schwann cells and the amplified region containing shRNA-inserted artificial miRNA and polyA signal were successively inserted into subcloning vector. A DNA fragment (~2.7-kb) containing all nucleotide units was digested from the vector backbone with EcoRI and PstI, purified, and injected into fertilized C57BL/6J oocytes, resulting in acquiring 2 founders. The original names (lines C and B) have been now designated as line #1 and line #2, respectively. Transgenic founder mice and established transgenic mice were routinely identified by tail DNA׳s genomic PCR with primers 1 and 2 (5′-ATGGTGAGCAAGGGCGAGGAGCTG-3′ and 5′-CTTGTACAGCTCGTCCATGCCGAGAGTGATC-3′, respectively). They were also identified by Southern blotting with BamHI-digested tail DNA hybridized to a radioisotope-labeled genomic probe specific for the transgene [1]. The transgenic allele resulted in a hybridized band of ~1.0 kb and the hybridized band was compared with the copy number standard. The line #1 harbored 20 copies per allele [1] whereas the line #2 did 2 copies in this study.

### 1.2 Immunoblotting

**Fig. 1:**
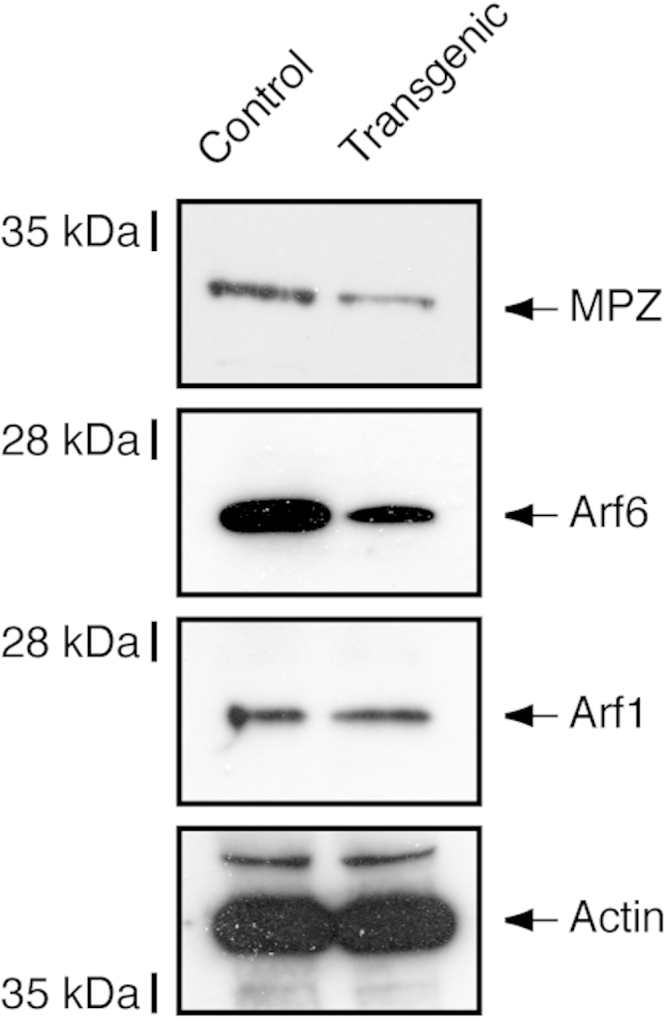
Expression of Arf6 in Arf6 shRNA transgenic mice. Transgenic mouse and littermate control mouse sciatic nerves at 7 days old (3 independent mice) were lysed and immunoblotted with an antibody against MPZ, Arf1 or 6, or actin. The representative immunoblots are shown.

Mouse sciatic nerves were lysed in lysis buffer (50 mM HEPES-NaOH, pH 7.5, 20 mM MgCl_2_, 150 mM NaCl, 1 mM dithiothreitol, 1 mM phenylmethane sulfonylfluoride, 1 μg/ml leupeptin, 1 mM EDTA, 1 mM Na_3_VO_4_, and 10 mM NaF) containing detergents (0.5% NP-40, 1%CHAPS, and 0.1%SDS). These detergents are important for myelin protein isolation [1]. Equal amounts of the proteins (20 µg of total proteins) in centrifuged cell supernatants were heat-denatured for immunoblotting by means of the TransBlot TurboTransfer System (Bio-Rad, Hercules, CA, USA). The transferred membranes were blocked with a Blocking One kit (Nacalai) and immunoblotted using primary antibodies (anti-Arf1 and anti-Arf6 [Santa Cruz Biotechnology], and MPZ and actin [MBL]), followed by peroxidase-conjugated secondary antibodies (Nacalai). The bound antibodies were detected using an ImmunoStar Zeta kit (Wako). In Arf6 shRNA transgenic mice, decreased expression levels of Arf6 and myelin marker MPZ were observed. In contrast, those of Arf1 and control actin were comparable in transgenic mice and littermate controls (Fig. 1).

### 1.3 Electron microscopy of mouse sciatic nerve

**Fig. 2:**
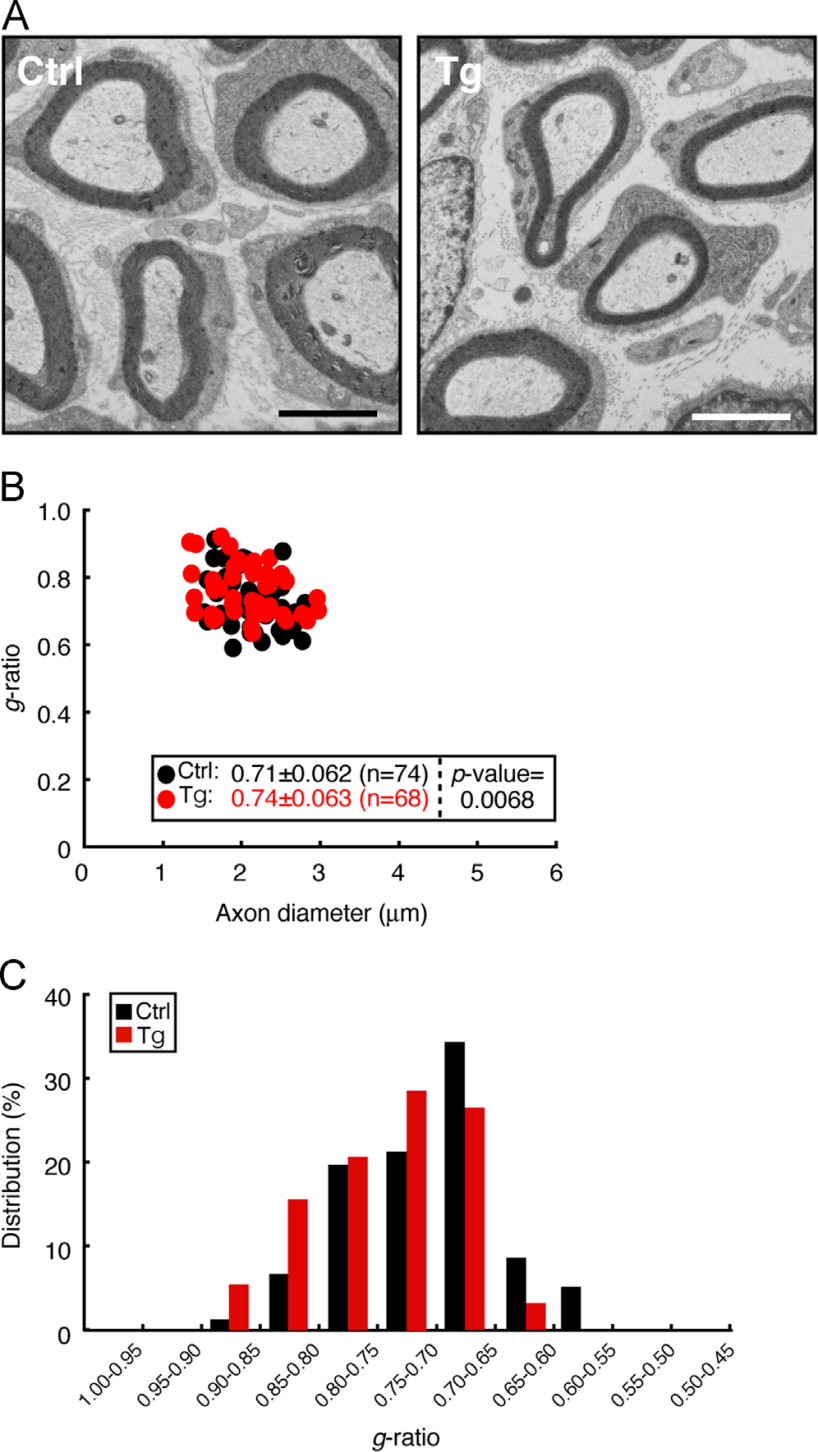
4-Day-old Arf6 shRNA transgenic mice exhibit decreased myelin thickness. (A) The representative electron micrographs of control (Ctrl) or transgenic (Tg) mouse sciatic nerve cross sections are shown. The scale bars indicate 2 µm. (B) The *g*-ratios are plotted for axon diameters. The average *g*-ratio and the *p*-value are also shown in the graph. The *n* number indicates total number of appreciably counted myelinated axons from 3 independent mice. (C) The data are presented in the form of the relative distributions of the *g*-ratios.

**Fig. 3:**
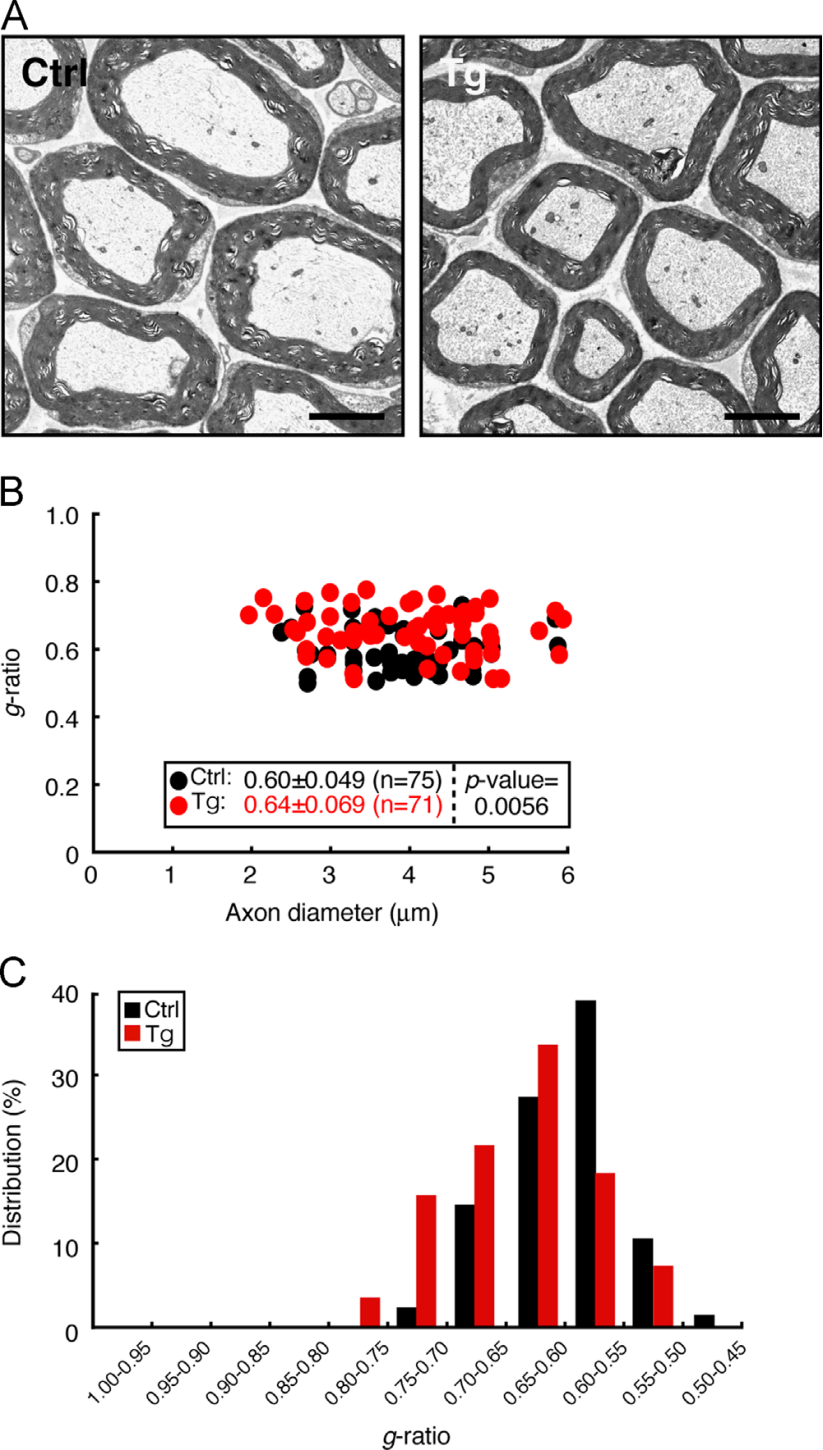
60-Day-old Arf6 shRNA transgenic mice also exhibit decreased myelin thickness. (A) The representative electron micrographs of control or transgenic mouse sciatic nerve cross sections are shown. The scale bars indicate 2 µm. (B) The *g*-ratios are plotted for axon diameters. The average *g*-ratio and the *p*-value are also shown in the graph. The *n* number indicates total number of appreciably counted myelinated axons from 3 independent mice. (C) The data are presented in the form of the relative distributions of the *g*-ratios.

Mouse sciatic nerves were fixed with 2% paraformaldehyde and 2% glutaraldehyde in 0.1% cacodylate buffer. The tissues were postfixed with buffered 2% osmium tetroxide, dehydrated with an ethanol gradient, treated with acetone, and embedded in epoxy resin. Ultrathin sections of cross sections were stained with uranyl acetate and lead citrate. They were observed and photographed with Hitachi or JEOL electron microscope system [1], [2]. Myelinated nerves in cross sections were randomly taken and the *g*-ratio, the numerical ratio of the axon diameter to the diameter of the outermost myelinated fiber of each axon, and the average were calculated. Thinner myelin sheaths yield larger *g*-ratios. 4-day-old transgenic mice exhibited decreased myelin thickness compared to littermate controls (0.74±0.063 in transgenic mice vs. 0.71±0.062 in controls, *p*=0.0068; Fig. 2). Decreased levels were not as much as those in the line #1 [1], likely depending on transgene copy number. While similar results were observed in 60-day-old transgenic mice (0.64±0.069 in transgenic mice vs. 0.60±0.049 in controls, *p*=0.0056; Fig. 3), the difference became smaller.

### 1.4 Mouse sciatic nerve staining

**Fig. 4:**
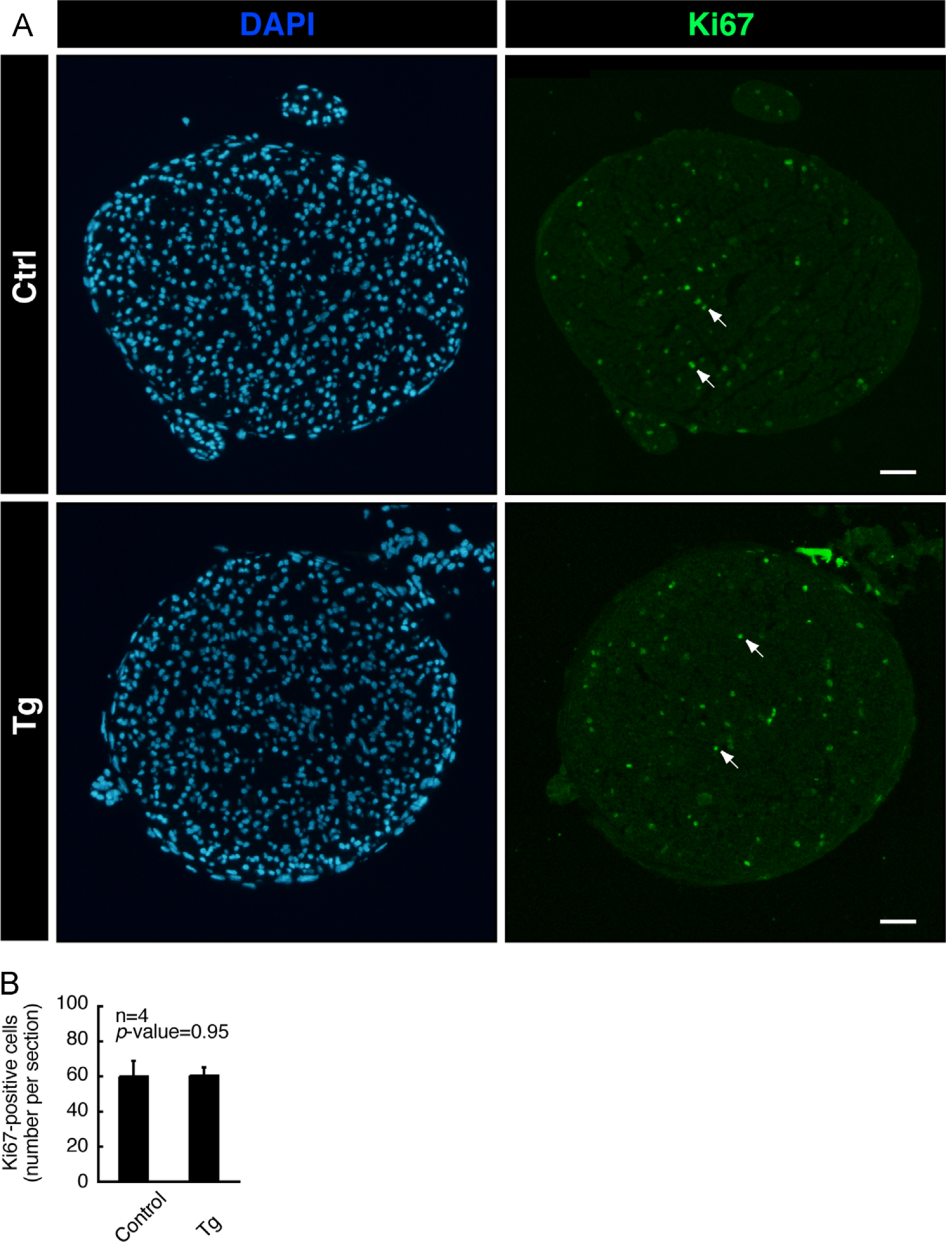
Ki67-staining. (A) In 7-day-old sciatic nerve cross sections of Arf6 shRNA transgenic and control mice, cells positive for the cell proliferation antigen Ki67 are shown in green fluorescence. The representative nuclear DAPI staining is also shown in blue fluorescence. Since sciatic nerve cross sections mainly contain Schwann cell bodies and nerve fibers, DAPI-staining nuclei are nearly identical with Schwann cell nuclei. The scale bars indicate 100 µm. The arrows indicate examples of Ki67-positive cells. (B) The number of Ki67-positive cells per section (*n*=4 independent mice) is shown in the graph.

**Fig. 5:**
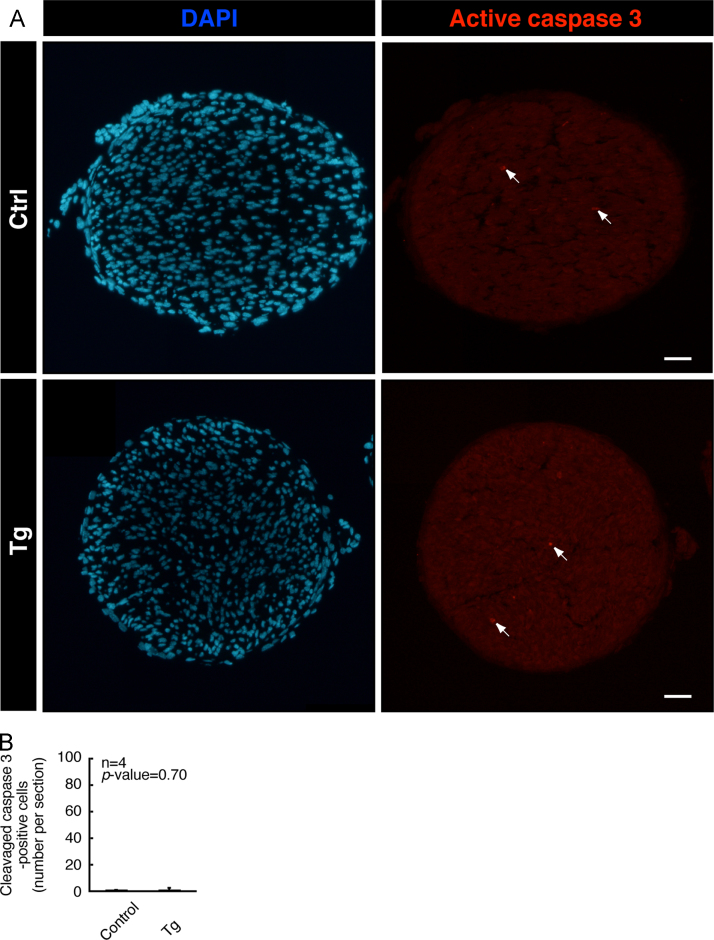
Active caspase 3-staining. (A) In 7-day-old sciatic nerve cross sections of Arf6 shRNA transgenic and control mice, cells positive for the apoptotic active caspase 3 are shown in red fluorescence. The representative nuclear DAPI staining is also shown in blue fluorescence. The scale bars indicate 100 µm. The arrows indicate examples of active caspase 3-positive cells. (B) The number of active caspase 3-positive cells per section (*n*=4 independent mice) is shown in the graph.

Tissues were perfused first with PBS and then with PBS containing 4% paraformaldehyde. They were postfixed with 4% paraformaldehyde, which was subsequently replaced with 20% sucrose, and embedded in Sakura Finetechnical Tissue-Tek reagent. Microtome sections were blocked, and incubated first with primary antibody (Cell Signaling Technology) against cell proliferation marker Ki67 or active caspase 3 and then with fluorescence-labeled secondary antibodies. The glass coverslips were mounted with DAPI-containing Vectashield. The fluorescent images were captured using DM2500 microscope system and analyzed using a LAS software (Leica) or captured using BX51 microscope system and analyzed using a DP2-BSW software (Olympus) [1], [2]. Ki67- or active caspase 3-staining was comparable in transgenic mice and controls (Fig. 4, Fig. 5). The respective *p*-values were not significant in Ki67- or active caspase 3-staining sections.

### 1.5 Statistical analysis

Data are presented as mean ± SD from independent experiments. Intergroup comparisons were performed using unpaired Student׳s *t* test. Differences were considered significant when *p* value is less than 0.01.
