# Supplementary material for: Data supporting Arf6 regulation of Schwann cell differentiation and myelination
Source: Data Brief. 2015 Oct 3;5:388–95. doi: 10.1016/j.dib.2015.09.025 (PMC4773367; doi:10.1016/j.dib.2015.09.025)
Supplement: Supplementary file 1 — Supplementary material [file mmc1.zip › coi_disclosure5.pdf]

è-¦å‘Š : ã•“ã•®ãƒ•ã‚©ãƒ¼ãƒ ã•¯ã€•ç•¾åœ¨ã•®ãƒ•ãƒ¼ã‚¸ãƒ§ãƒ³ã•® Acrobat ã•¾ã•Ÿã•¯ Adobe Reader ã•§ã•¯ã‚µãƒ•ãƒ¼ã

å®Œå…¨ã•ªã‚µãƒ•ãƒ¼ãƒˆã•Œå¿…è¦•ã•ªå ´å•ˆã•¯ã€•æœ€æ–°ãƒ•ãƒ¼ã‚¸ãƒ§ãƒ³ã•«ã‚¢ãƒƒãƒ—ã‚°ãƒ¬ãƒ¼ãƒ‰ã•—ã•¦ã••ã•
